# The Superoanterior Fasciculus (SAF): A Novel White Matter Pathway in the Human Brain?

**DOI:** 10.3389/fnana.2019.00024

**Published:** 2019-03-05

**Authors:** Szabolcs David, Anneriet M. Heemskerk, Francesco Corrivetti, Michel Thiebaut de Schotten, Silvio Sarubbo, Francesco Corsini, Alessandro De Benedictis, Laurent Petit, Max A. Viergever, Derek K. Jones, Emmanuel Mandonnet, Hubertus Axer, John Evans, Tomáš Paus, Alexander Leemans

**Affiliations:** ^1^Image Sciences Institute, University Medical Center Utrecht and Utrecht University, Utrecht, Netherlands; ^2^Department of Neurosurgery, Lariboisière Hospital, APHP, Paris, France; ^3^Centre for Neuroimaging Sciences, Institute of Psychiatry, King’s College London, London, United Kingdom; ^4^Structural and Functional Connectivity Lab Project, Department of Emergency, Division of Neurosurgery, “S. Chiara” Hospital, Azienda Provinciale per i Servizi Sanitari (APSS), Trento, Italy; ^5^Department of Neurosciences, Division of Neurosurgery, “Bambino Gesù” Children Hospital, IRCCS, Rome, Italy; ^6^Groupe d’Imagerie Neurofonctionnelle (GIN), Institut des Maladies Neurodégératives (IMN)-UMR5293-CNRS, CEA, Université de Bordeaux, Bordeaux, France; ^7^Cardiff University Brain Research Imaging Centre, School of Psychology, Cardiff, United Kingdom; ^8^Hans Berger Department of Neurology, Jena University Hospital, Friedrich-Schiller University Jena, Jena, Germany; ^9^Bloorview Research Institute, Holland Bloorview Kids Rehabilitation Hospital, Toronto, ON, Canada; ^10^Departments of Psychology and Psychiatry, University of Toronto, Toronto, ON, Canada

**Keywords:** brain, diffusion MRI, fiber tractography, dissection, polarized light imaging, neuroanatomy, superoanterior fasciculus

## Abstract

Fiber tractography (FT) using diffusion magnetic resonance imaging (dMRI) is widely used for investigating microstructural properties of white matter (WM) fiber-bundles and for mapping structural connections of the human brain. While studying the architectural configuration of the brain’s circuitry with FT is not without controversy, recent progress in acquisition, processing, modeling, analysis, and visualization of dMRI data pushes forward the reliability in reconstructing WM pathways. Despite being aware of the well-known pitfalls in analyzing dMRI data and several other limitations of FT discussed in recent literature, we present the superoanterior fasciculus (SAF), a novel bilateral fiber tract in the frontal region of the human brain that—to the best of our knowledge—has not been documented. The SAF has a similar shape to the anterior part of the cingulum bundle, but it is located more frontally. To minimize the possibility that these FT findings are based on acquisition or processing artifacts, different dMRI data sets and processing pipelines have been used to describe the SAF. Furthermore, we evaluated the configuration of the SAF with complementary methods, such as polarized light imaging (PLI) and human brain dissections. The FT results of the SAF demonstrate a long pathway, consistent across individuals, while the human dissections indicate fiber pathways connecting the postero-dorsal with the antero-dorsal cortices of the frontal lobe.

## Introduction

Fiber tractography (FT) based on diffusion magnetic resonance imaging (dMRI; Jeurissen et al., 2017) is widely used for investigating microstructural properties of white matter (WM) fiber-bundles (Alexander et al., 2017), and for mapping structural connections of the human brain (Wakana et al., 2004; Sotiropoulos and Zalesky, 2017). Since the first endeavors of FT (Basser et al., 1994, 2000; Conturo et al., 1999; Jones et al., 1999; Mori et al., 1999; Catani et al., 2002), many studies have contributed to the improvement of data quality and the level of detail. Recent advances in MRI hardware and acquisition (Anderson, 2005; Moeller et al., 2010; Setsompop et al., 2013, 2018; Sotiropoulos et al., 2013; Andersson et al., 2016) have improved significantly dMRI data in terms of spatial resolution, angular resolution signal-to-noise ratio, and geometrical distortion reduction. Additionally, notable developments in data processing have reduced errors from the individuals’ head motion (Leemans and Jones, 2009), data outliers (Chang et al., 2005; Pannek et al., 2012; Collier et al., 2015; Tax et al., 2015), eddy currents (Andersson and Sotiropoulos, 2015, 2016; Andersson et al., 2016), EPI distortions (Andersson et al., 2003), Gibbs-ringing (Perrone et al., 2015; Kellner et al., 2016; Veraart et al., 2016), and other data artifacts, like drift of the diffusion signal (Vos et al., 2017). Besides the traditional diffusion tensor model, more sophisticated methods have been developed to resolve crossing fibers (Lin et al., 2003; Tuch, 2004; Tournier et al., 2004; Wu and Alexander, 2007; Dell’Acqua et al., 2013; Tax et al., 2014; Jensen et al., 2016). Together, all these improvements allow for more reliable FT results, thereby resolving complex fiber architecture, smaller branches of fiber bundles or minor fiber-pathways (Tournier et al., 2008). Although dMRI based tractography is a promising technique, there are many well-known pitfalls and limitations in acquiring and analyzing dMRI data (Jones and Cercignani, 2010; Jones et al., 2013b; O’Donnell and Pasternak, 2015). Thus, dMRI is an indirect approach for measuring the underlying WM pathway properties, as it is an approximation of the average diffusion within a given voxel. Modeling the diffusion results in a proxy of main directions, and thus the exact architecture of the pathways cannot be determined unambiguously. The modeling errors can cause tracts to stop prematurely or jump from one WM structure to another, resulting in false negative or false positive connections. The strength of connectivity, when assessed *via* probabilistic FT, is unreliable due to its sensitivity to data quality (Mesri et al., 2016).

Despite recent efforts to increase the accuracy of FT by either pruning the tractograms using anatomical information (Smith et al., 2012; Roine et al., 2015) or by including microstructural information to disambiguate between pathways (Smith et al., 2013), the International Society for Magnetic Resonance in Medicine (ISMRM) Tractography Challenge in 2015 demonstrated that some data-processing pipelines could result in large errors as the reconstructed tracts produced an average ratio of 1:4 in false positive-false negative connections and 45% in bundle overlap when compared with pre-defined, ground-truth streamlines (Maier-Hein et al., 2017).

In another study, the sensitivity (true connections) and specificity (avoidance of false connections) of dMRI tractography was investigated by combining tracer studies and high quality dMRI data (Thomas et al., 2014). This research showed a sensitivity-specificity trade-off, i.e., the number of true connections increased simultaneously with the number of false connections. Additionally, the anatomical accuracy of the tracts depended on the studied pathway, the acquisition and the processing pipeline. This stresses the fact that comparing results across studies can suffer from the differences in acquisition settings and processing methods. Therefore, reconstructing fiber pathways from dMRI FT should be performed with great care and additional support from other methods is welcome.

Nonetheless, being aware of the recognized issues, we present the description of a novel fiber tract, the superoanterior fasciculus (SAF), in the frontal lobe which—to the best of our knowledge—has not been documented before with dMRI based tractography with this level of detail. The tract is slightly curved and follows the same arc profile of the cingulum bundle, but is located more frontally and above the frontal part of the cingulum; it can be found in both hemispheres.

Traditionally, the description of new WM fiber bundles went through several stages. For example, WM histology and bundle degeneration studies (Dejerine and Dejerine-Klumpke, 1895) and dissection (Ludwig and Klingler, 1956; Heimer, 1983) studies supported the presence of a structure in *post mortem* brains, which was confirmed later *via* dMRI-based FT in the living human brain. Conturo et al. (1999) laid the foundations for selection and analysis of tracts based on “region of interest (ROI)”, which opened up a new era of tract-based investigations from neuroscience (Lebel et al., 2008; Thiebaut de Schotten et al., 2011a, 2012) to clinical applications (Thiebaut de Schotten et al., 2005; Bartolomeo et al., 2007; Deprez et al., 2012). The access to large samples made the construction of *in vivo* WM tract atlases and guidelines feasible (Mori et al., 2005; Wakana et al., 2007; Hua et al., 2008). In this work, we reconstruct the proposed structure using specific ROIs along with modeling of the diffusion MR signal based on constrained spherical deconvolution (CSD; Tournier et al., 2004; Tournier et al., 2007).

To minimize the possibility that these findings are based on acquisition or processing artifacts, we used datasets from different projects: young Adult human connectome project (HCP; Glasser et al., 2013; Sotiropoulos et al., 2013; Van Essen et al., 2013), Avon Longitudinal Study of Parents and Children (ALSPAC; Golding, 2004), Multiple Acquisitions for Standardization of Structural Imaging Validation and Evaluation (MASSIVE; Froeling et al., 2017), an in-house dataset (Jeurissen et al., 2011), and robust processing pipelines to show these trajectories. While the main focus of this research was based on dMRI, we complemented our findings with other non-MRI techniques, such as polarized light imaging (PLI) microscopy (Axer et al., 2011) and human brain dissections. Preliminary results of this work on the SAF have been presented at the 2015 ISMRM meeting in Toronto, ON, Canada (Heemskerk et al., 2015).

## Materials and Methods

In the methods section, we describe first the datasets that were used for the investigation. We then explain the dMRI processing, FT and analysis steps. Finally, we describe the PLI and dissection methods.

### Diffusion MRI

#### Datasets

Four dMRI datasets acquired on different platforms were used to study the tract. The datasets represent a large diversity in data quality and number of samples, which helps to generalize the conclusions.

##### Dataset 1

Minimally processed dMRI data from the HCP S500 release were used. Briefly, the data were acquired with a 1.25 mm isotropic voxel size; three shells with b = 1,000, 2,000 and 3,000 s/mm^2^ in 90 DW volumes and six non-weighted images per shell. Every participant for whom all the 90 b = 1,000 s/mm^2^ and 90 b = 3,000 s/mm^2^ images were available along with all 18 b = 0 s/mm^2^ volumes, and were not listed among participants with known anatomical anomalies or data quality issues were included in the analysis. The IDs of the excluded participants are listed on the HCP wiki page (HCP wiki, 2018). The selection resulted in 409 healthy participants (243 females and 166 males), between the age of 22 and 36 years. Low b-value images were analyzed with DTI and CSD models separately using 90 b = 1,000 s/mm^2^ and nine unweighted volumes, while the higher b-shell was processed with CSD only using 90 b = 3,000 s/mm^2^ and 18 unweighted volumes. For a subset of 10 participants we also analyzed the b = 1,000 s/mm^2^, 2,000 s/mm^2^ and 3,000 s/mm^2^ shells with both CSD and DTI models.

##### Dataset 2

Ten dMRI datasets were used from the ALSPAC cohort, each consisting of 30 diffusion gradients with b = 1,200 s/mm^2^, and 2.4 mm isotropic voxel size as described by Björnholm et al. (2017).

##### Dataset 3

The dataset is described in the work of Jeurissen et al. (2011). In summary, one participant’s dMRI consisted of 60 diffusion directions, 2.4 mm isotropic voxel size (resampled to 1 mm isotropic) and b = 3,000 s/mm^2^.

##### Dataset 4

The dataset from the MASSIVE (Froeling et al., 2017) acquisition was used. In short, we used a subset of 22 b = 0 s/mm^2^ volumes and 250 b = 3,000 s/mm^2^ volumes and an isotropic resolution of 2.5 mm^3^ of one participants.

#### Image Processing and Signal Modeling

All datasets were preprocessed with *ExploreDTI* v.4.8.6 (Leemans and Jones, 2009; Leemans et al., 2009), except for the HCP dataset, which was already preprocessed (see Dataset 1 below). Further modeling (DTI and CSD) and defining ROI configurations were also performed with *ExploreDTI*.

##### Dataset 1

Briefly, *FSL* (Jenkinson et al., 2012) tools *topup* (Andersson et al., 2003) and *eddy* (Andersson and Sotiropoulos, 2016) were used to correct for head motion and geometrical distortions arise from eddy currents and susceptibility artifacts. Finally, the DWIs were aligned to the structural T1 image. For the full description, see the work of Sotiropoulos et al. (2013). DTI estimation of the low b-value shell for the full population was performed using REKINDLE (Tax et al., 2015). The fiber orientation distribution (FOD) in each voxel was estimated using CSD (Tournier et al., 2004, 2007) with the recursive calibration method (peak ratio threshold = 0.01; Tax et al., 2014) with maximum harmonic degree of L_max_ = 8, in the high and low b-value shells separately. For a subset of 10 subjects, we performed additional analyses to test the effects of b-value and choice of diffusion modeling. For this, we used the following settings: (a) CSD with b = 2,000 s/mm^2^; (b) CSD with b = 1,000 s/mm^2^; (c) CSD with L_max_ = 6; (d) CSD with calibration of the response function based on FA > 0.8; and (e) DTI estimation using REKINDLE.

##### Dataset 2

Motion-distortion correction and Gaussian anisotropic smoothing (Van Hecke et al., 2010) were performed with *ExploreDTI*. The FOD in each voxel was estimated using CSD with recursive calibration (peak ratio threshold = 0.01) and L_max_ = 8.

##### Dataset 3

Motion-distortion correction was performed with *ExploreDTI*. The dataset was resampled to 1 mm isotropic voxel size to increase the level of detail (Dyrby et al., 2014). FODs were estimated using CSD with recursive calibration (peak ratio threshold = 0.01), L_max_ = 8.

##### Dataset 4

Dataset 4 was motion, distortion corrected and resampled to 1 mm isotropic voxel size. Similar to the previous datasets, the FOD was estimated using CSD with recursive calibration (peak ratio threshold = 0.01), L_max_ = 8.

#### Fiber Tractography

For all four datasets, the deterministic FT framework of Jeurissen et al. (2011) was used for the CSD approach, with the following parameter settings: FOD threshold = 0.1, angle deviation = 45°, step size = 1 mm and minimal tract length = 20 mm. Whole-brain FT was performed with uniform distribution of seed points defined at a 2 mm resolution. For DTI based FT, the settings are: step size = 1 mm, minimal tract length = 20 mm, angle deviation = 45°, FA seed and tracking threshold of 0.2 and uniform seed point resolution of 2 mm.

#### ROI Configuration for Reconstructing the SAF

**Figure 1:**
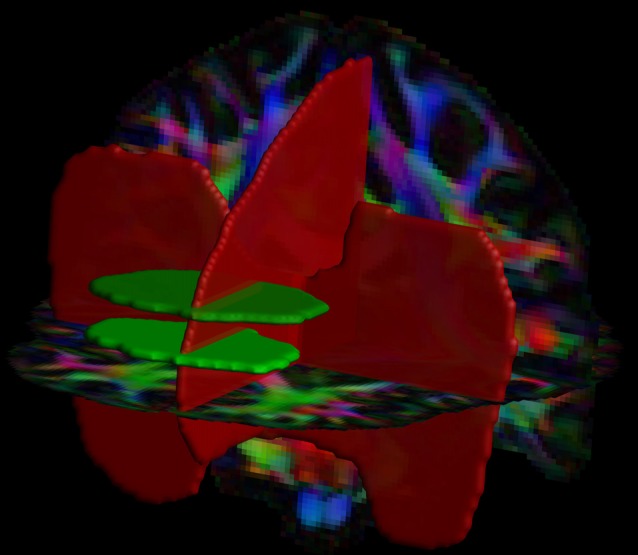
Positions of the two AND (in green) and two NOT (in red) regions of interest (ROIs) are shown with a direction encoded color (DEC)-FA map. This ROI configuration was used for the tract selection of the superoanterior fasciculus (SAF) in both hemispheres.

Automated, large-scale tract selection on the HCP dataset was obtained by atlas-based tractography segmentation (Lebel et al., 2008). On the template dataset, we defined 4 Boolean “AND” and “NOT” ROIs: 2 axial AND, 1 sagittal NOT and 1 coronal NOT ROIs (Figure 1). The 2 axial AND ROIs were placed as follows: the first one was located at the height of approximately half the genu of the corpus callosum (CC) and the second ROI was placed 10 slices (equals to 12.5 mm in the HCP dataset) superior. Both AND ROIs included the medial frontal area and excluded the cingulum. A NOT ROI was placed midsagittal to exclude fiber pathways from the CC. Additionally, one coronal NOT ROI was placed posterior of the frontal lobe and below the CC to exclude fibers from the inferior fronto-occipital fasciculus (iFOF).

#### Consistency Evaluation of the SAF

##### Within Cohort

For each of the 409 participants from the HCP cohort, the visitation mask of the reconstructed SAF was normalized to the Montreal Neurological Institute (MNI) template (Fonov et al., 2011), using the transform functions provided by the HCP team. The resulting map was thresholded at 1 and binarized. The group map displays the fraction of participants for whom the tract mask of the SAF is present in each voxel of the MNI space.

##### Across Acquisition Protocols

The tractography results of participants from the four different datasets were compared to investigate the consistency of the SAF configuration across different acquisition methods.

##### Across Processing Settings

We evaluated the effects of different processing settings on the tractography results for 10 subjects of the HCP dataset.

### Polarized Light Microscopy

PLI is a microscopy method that uses the birefringent properties of myelin sheath to quantify the main fiber orientation in histological sections (Larsen et al., 2007). In this work, we used data previously involved in the investigation of the anterior cingulum (Axer et al., 2011). One of the studied brains was sectioned as to expose the area of interest but was not investigated previously. For details on acquisition and processing see the corresponding study (Axer et al., 2011). Briefly, a 4% aqueous formalin-fixed human brain was macroscopically dissected and a 1.5 cm thick slab of the medio-frontal brain including the anterior cingulum bundle was cut in four blocks. Each of these four blocks were serially sliced and analyzed. Sections were obtained with a cryostat microtome (CM3050 S, Leica Microsystems, Bensheim, Germany) at a thickness of 100 μm. Aqueous mounting medium Aquatex^TM^ (Merck, Darmstadt, Germany) was used for mounting. The histological sections were placed between two coupled crossed polars which can be rotated. Birefringence in the tissue is able to twist some of the light so that it can pass through the second polarizer and be imaged. The orientation of the nerve fibers influences the transmission of plane-polarized light at different azimuths. A CCD camera (Axiocam HR, Carl Zeiss, Göttingen, Germany, basic resolution of 1,388 × 1,040 pixel) was used to capture the light. The brain slices were digitized under azimuths from 0 to 80° using two polars only. These sequences were used to estimate the inclination of fibers (in z-direction). The same slices were digitized under azimuths from 0 to 160° in steps of 20° using a quarter wave plate additionally. These sequences were used to estimate the direction of the fibers in xy-direction.

### Dissection (Paris)

Post-mortem brain dissection was performed in the laboratory of neuroanatomy of Lariboisière Hospital in Paris, France by two neurosurgeons (EM, FCorr).

Five human cerebral hemispheres, obtained from fresh autopsy, were fixed in 10% formalin solution for at least 3 weeks. These specimens were frozen at −18°C for 2 weeks and then unfrozen at room temperature. This process (Klingler technique’s; Klingler, 1935) induces water crystallization in the brain tissue that, by spreading along the WM fibers, facilitates visualization and dissection of the subcortical WM fibers.

The dissection was performed with light microscopy (OPMI pico, Carl Zeiss, Inc., Oberkochen, Germany) and video recorded with a high-resolution digital camera (Karl Storz GmbH, Tuttlingen, Germany).

Initial observation of the configurational shape of sulci and gyri of the mesial cortical surface of frontal lobe was always performed before dissection. The dissection always started from the cingulate fibers extending to the entire mesial and lateral surface of the frontal lobe.

### Dissection (Trento)

Dissections below were performed by three neurosurgeons (SS, FCors, and AB) in the context of the Structural and Functional Connectivity Lab (SFC-Lab) Project, which was approved by the Ethical Committee of the Azienda Provinciale per i Servizi Sanitari (APSS) of Trento, Italy.

Three right hemispheres were prepared for Klingler’s dissection (Klingler, 1935) according to the protocol previously reported (Sarubbo et al., 2015; De Benedictis et al., 2014, 2016). It starts with an injection of formalin 10% in carotids and vertebral arteries, then an immersion in formalin 10% for 40 days, and finally the progressive freezing at −80° and de-freezing procedure. After the first de-frost process and removal of arachnoids and vessels, we started the micro-dissection under microscope with 4× magnification with wooden spatulas, approaching at the medial and ventral WM of the frontal lobe from the latero-dorsal cortical surface and leaving intact the gray matter at the tip of the gyri exposed to highlight the territories of terminations.

## Results

### Tract Description

**Figure 2:**
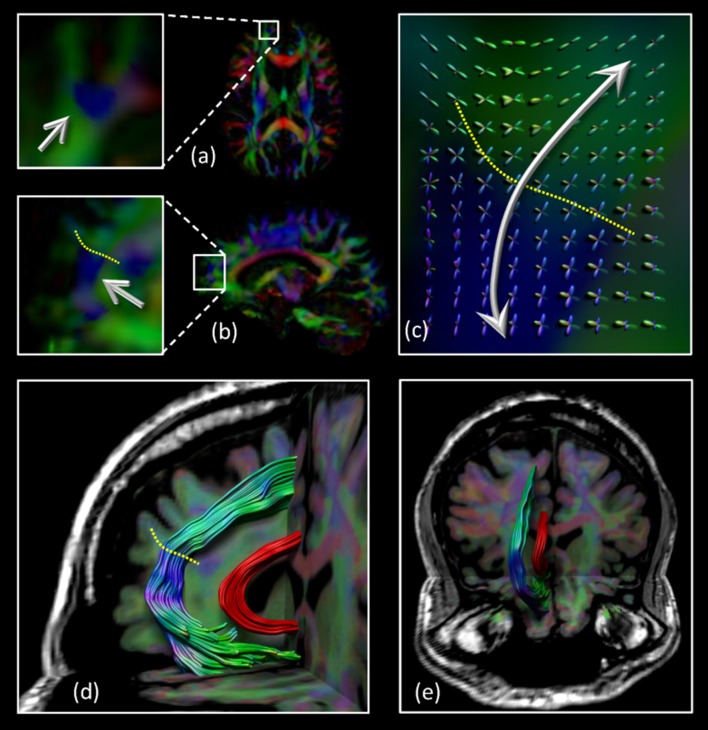
Location of the SAF: the axial **(A)** and sagittal **(B)** view of part of the region that intersects the SAF (blue region indicated by the arrows). The complex fiber architecture in this area can be appreciated from the fiber orientation distributions (FODs) overlaid on the sagittal view in **(C)** with the dotted yellow line identifying the interface between regions with different dominant fiber populations (“blue” vs. “green” on the DEC-FA map). Sagittal **(D)** and coronal **(E)** views of the SAF configuration. For displaying purposes, only one of the bilateral SAFs is shown. The cingulum bundle is shown in red to provide anatomical reference. Grayscale background is the T1-weighted magnetic resonance imaging (MRI) of the subject.

The architectural configuration of the SAF is shown in Figure 2 where dataset 3 was used with CSD based FT. The complex fiber architecture in the frontal area can be appreciated from the FODs overlaid on the sagittal view in Figures 2A–C with the dotted yellow line identifying the interface between regions with locally different dominant fiber populations [“blue” vs. “green” on the principal direction encoded color (DEC) map]. A medial and frontal view of the bilateral tract configuration is given in Figures 2D,E, respectively. Note that the SAF resembles the trajectory of the cingulum bundle (in red) but it is located more anteriorly, superiorly, and laterally.

**Figure 3:**
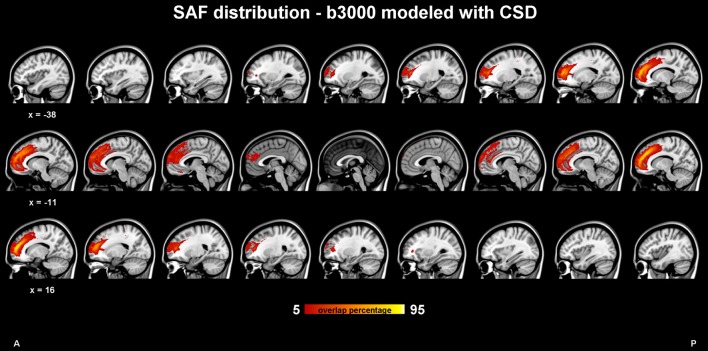
SAF probability map in 1 mm Montreal Neurological Institute (MNI) stereotaxic space from 409 subjects of the Human Connectome Project (HCP) cohort in sagittal view. Constrained spherical deconvolution (CSD) modeling with recursive calibration was used on the high b-value data. The % indicates the fraction of subjects for which the tract is present in a given voxel.

The SAF group composite map of the HCP participants (dataset 1) using CSD modeling with b = 3,000 s/mm^2^ is shown in Figure 3. Again, the bilateral tracts follow a similar trajectory as the cingulum, but more frontally [i.e., in front of the cingulate sulcus or within the superior frontal gyrus (SFG)] and slightly more laterally. The SAF pathways appear to spread from the rostrum of the CC to the ascending ramus of the cingulate sulcus and are medial to the corona radiata. Supplementary Figures S1, S2 show the population level map using the same ROIs, but with the 1,000 s/mm^2^ b-value dataset and modeled with DTI and CSD, respectively.

### Tract Consistency

#### Across Participants

**Figure 4:**
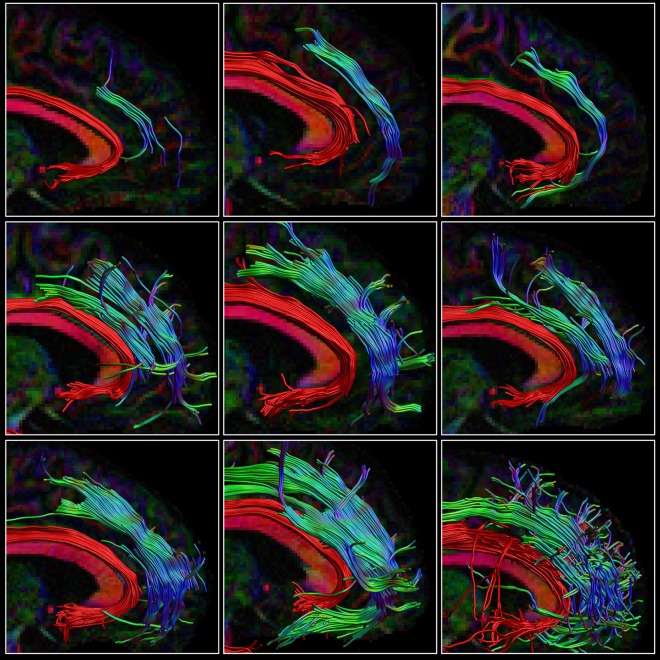
Consistency within the HCP cohort. The right SAF and cingulum is depicted in sagittal view for nine subjects from the HCP dataset highlighting the large variability in extent of the SAF. The cingulum is shown in red to provide anatomical reference. Tracts are plotted on top of the DEC-FA map.

The percentage overlap map in Figure 3 and Supplementary Figure S2 show that the main portion of the SAF is present for 90% of the participants, which is in the same range as other tracts (Thiebaut de Schotten et al., 2011b). The extent of the reconstructed SAF varied substantially across participants (see Figure 4). For some, a very extended and broad structure was found (Figure 4, bottom row), while in others we found only a narrow portion of the SAF (Figure 4, top row). The length of the streamlines also varied across participants, but also within the same individual. It is often observed that streamlines stop abruptly due to the absence of a corresponding FOD peak larger than the predefined threshold.

#### Across Data Acquisitions

**Figure 5:**
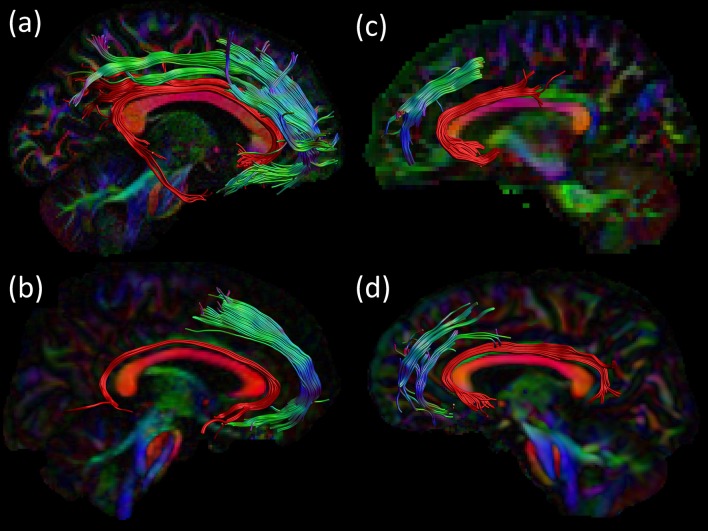
Consistency across acquisition protocols. For all four datasets an example of the SAF configuration is shown in sagittal view: right SAF and right cingulum for **(A)** dataset 1 and **(B)** dataset 3. Left SAF and left cingulum is shown for **(C)** dataset 2 and **(D)** dataset 4. The cingulum is shown in red to provide anatomical reference. Tracts are plotted on top of the DEC-FA map.

**Figure 6:**
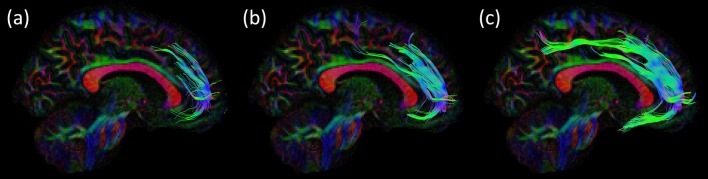
Consistency of the SAF configuration across acquisition strategies for the same subject of the HCP cohort using CSD based fiber tractography (FT). The effect of using different b-values is shown in the sagittal view, other settings are identical: **(A)** b = 1,000 s/mm^2^; **(B)** b = 2,000 s/mm^2^ and **(C)** b = 3,000 s/mm^2^. Trajectories of the SAF are plotted on top of the DEC-FA map.

Figure 5 reveals that the SAF was present in all samples that we analyzed. This observation excludes the possibility that the SAF is merely a scanner-specific artifact. While there are differences in acquisition settings between the different acquisition sites, there is no impact on the extent of the SAF. An example is shown in Figure 6, where the effect of using different b-values for the data acquisition on the SAF reconstruction is depicted. Low b-values result in less complex FODs leading to shorter and fewer streamlines, but CSD based modeling can still reveal the SAF configuration. CSD based tract coverage from low b-value data shows large overlap with the high b-value based CSD tracts, revealing that proper modeling of crossing fibers is imperative for reconstruction of the SAF. This can be appreciated as well in the population map in Supplementary Figure S2, in which DTI based FT cannot deal with complex fiber pathway configurations.

#### Across Processing Strategies

**Figure 7:**
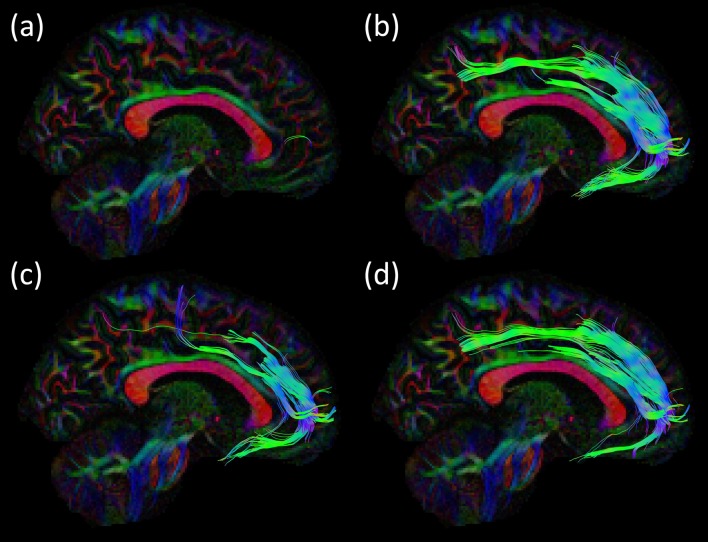
Consistency of the SAF configuration across different modeling and processing strategies for the same subject of the HCP cohort. In **(A)**, the DTI model was used, resulting in only few and likely spurious tract pathways, whereas the CSD models in **(B–D)** produce similar and plausible SAF trajectories, but with differences due to processing settings: **(B)** recursive calibration and L_max_ = 8; **(C)** recursive calibration and L_max_ = 6; and **(D)** FA calibration and L_max_ = 8. Tracts are plotted on top of the DEC-FA map and shown sagittal.

Modeling and processing steps also affected the extent of the reconstructed SAF (Figure 7). DTI analysis (Figure 7A) resulted in either no pathways at all or in some spurious ones. CSD-based modeling showed either shorter SAF pathways (L_max_ = 6, Figure 7C) or minor variations (FA calibration, Figure 7D). According to the population-level maps in Supplementary Figure S1, DTI-based reconstructions of the SAF cover only the most frontal parts of the tract, which is also depicted in Figure 2 with the blue color. In this location of the SAF, the locally dominant direction of diffusion is tangential with the SAF, irrespective of any other crossing pathways. Clearly the biggest impact on showing the SAF configuration was the modeling of crossing fibers, in our example using CSD over DTI.

### PLI

**Figure 8:**
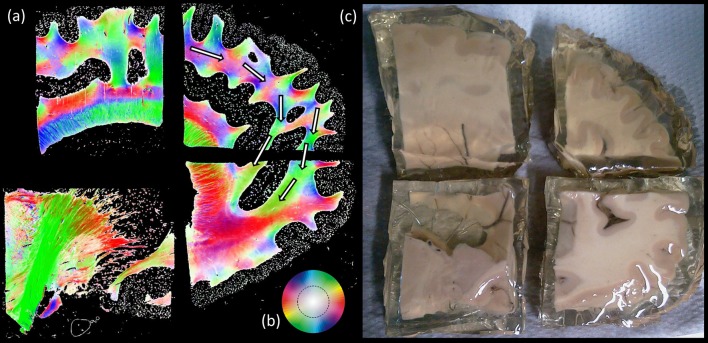
Polarized light imaging (PLI) results. **(A)** Four separate blocks of the same brain sliced in sagittal plane, where the fiber orientation maps are depicted correspond to the **(B)** color scheme circle. White arrows are indicating the location of the proposed pathway. Part **(C)** shows an example of formalin fixed human cadaver brain.

Figure 8 shows the ensemble of the four blocks color-coded by the main in-plane orientation per voxel. In the ROI of where the SAF is expected, a variety of colors and therefore locally dominant orientations were present. Nonetheless, a similar shape (indicated by the white arrows) as with tractography can be appreciated.

### Dissection (Paris)

**Figure 9:**
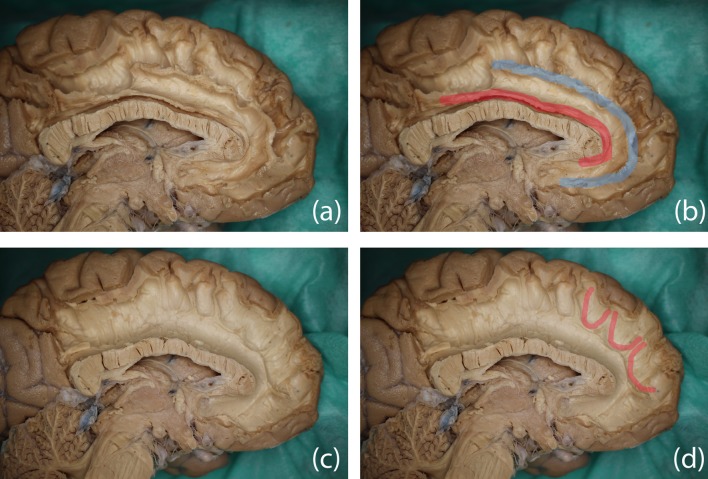
Dissection results in two different hemispheres and color overlays representing the proposed structure. Top row: **(A)** the original image and **(B)** possible trajectory of the SAF overlaid in blue, the cingulum is indicated in red as the anatomical reference. Bottom row: **(C)** original image and **(D)** several U-shaped fibers highlighted in red, which may form part of the SAF *via* FT.

We were able to isolate, in four of the five specimens, a series of fibers spreading over from the cingulate fibers that seemed to have the same anteroposterior orientation as the FT results of the SAF. These fibers were located above the cingulum in correspondence with the frontal gyrus (Figure 9). The careful dissection of the fiber complex revealed several different fibers: U-shaped fibers from cingulate, U-shaped fibers belonging to SFG and more lateral fibers from corona radiata (Figure 9). All of them had a vertical orientation; therefore, no correspondence to the anteroposterior orientation of the putative SAF.

### Dissection (Trento)

**Figure 10:**
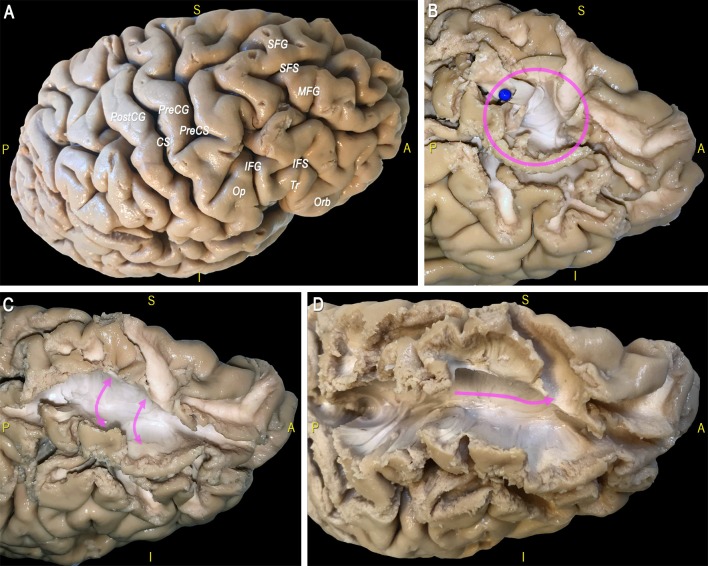
**(A)** The main sulci and gyri of the lateral frontal region of a right hemisphere are indicated, after removal of vessels, pia mater, and arachnoid membranes. **(B)** Decortication of the SFG and MFG allows identifying the U fibers under the SFS (blue pin, pink circle). **(C)** Exposition of U-fibers between di SFG and MFG (pink arrows). **(D)** Exposition of a thin layer of fibers with an antero-posterior course under the SFS (pink arrow). CS, central sulcus; IFG, inferior frontal gyrus; IFS, inferior frontal sulcus; MFG, middle frontal gyrus; Op, opercular part of the IFG; Orb, orbital part of the IFG; PreCG, precentral gyrus; PreCS, precentral sulcus; PostCG, postcentral gyrus; S, superior; SFS, superior frontal sulcus; SFG, superior frontal gyrus; Tr, triangular part of the IFG.

After exploration of the sulco-gyral anatomy (Figure 10A) we proceeded with a gentle peel out of the gray matter of the depth and lateral surface of all the gyri of the dorsal portion of the frontal and parietal lobes (Figure 10B). Then we removed the U-fibers connecting the most anterior thirds of the middle and superior frontal gyri, at the level of the superior frontal sulcus (SFS; Figure 10C). After a progressive and cautious removal of the U-fibers connecting the lateral and medial cortices facing at the border of the SFS, we exposed a thin layer of fibers with an antero-posterior course (in all the three hemispheres dissected; Figure 10D). We followed back with a gentle micro-dissection this layer of fibers exposing the posterior territories of terminations within the dorsal third of the pre-central gyrus. Anteriorly we exposed this thin bundle with an arching course, that follows the physiologic curvature of the frontal anterior cortices, and we exposed the territories of terminations within: the frontal pole, the fronto-orbital cortex and the fronto-orbito-lateral cortex.

It was not possible to follow these fibers further back (i.e., behind the central sulcus) in any of the specimen because: (1) we did not find clear signs of continuity; and (2) the dense and intermingled crossing area with the vertical fibers ascending and descending to the pre- and post-central gyri.

**Figure 11:**
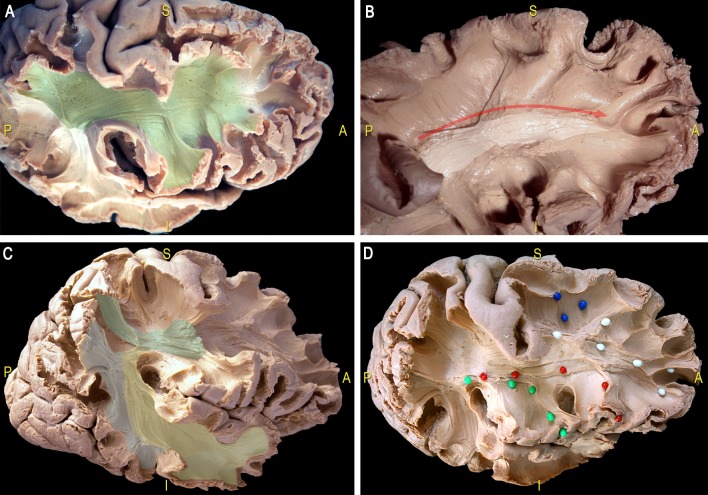
Dissection of a right hemisphere according to Klingler’s technique. **(A)** Exposition of the most ventral components of the anterior indirect component of the SLF (SLF II and SLF III; green area). **(B)** Thin layer of fibers connecting the postero-dorsal with the antero-dorsal cortices of the frontal lobe (white area, red arrow). **(C)** Dissection of the main components the SLF: indirect anterior (green area), indirect posterior (blue area); direct (yellow area). **(D)** U-fibers (blue pins), dorsal frontal fibers (white pins), frontal terminations of the AF, respectively in the ventral premotor cortex (green pins) and the pars opercularis of the IFG (red pins). AF, arcuate fasciculus; SLF, superior longitudinal fascicle.

This thin layer of fibers is located in the most medial and dorsal portion of frontal WM and connects the postero-dorsal with the antero-dorsal cortices of the frontal lobe (Figure 11A). We performed also the micro-dissection of the most ventral components of the superior longitudinal fascicle (namely, SLF II and III; Figures 11B–D), accordingly to the recent description by Fernández-Miranda et al. (2015); these SLF components occupy the medio-dorsal and ventral WM of the frontal lobe and are clearly distinguishable from the SAF fibers by location, direction and course.

## Discussion

By taking advantage of advanced dMRI methodology, we have identified a consistent bilateral pathway, referred to as the SAF, *via* FT in the frontal lobe, which has not been described before (Catani et al., 2012; Makris et al., 2005; Thiebaut de Schotten et al., 2011b). Reproducibility across multiple participants, different data samples and acquisition settings boosted our confidence that this finding is not based on imaging artifacts.

**Figure 12:**
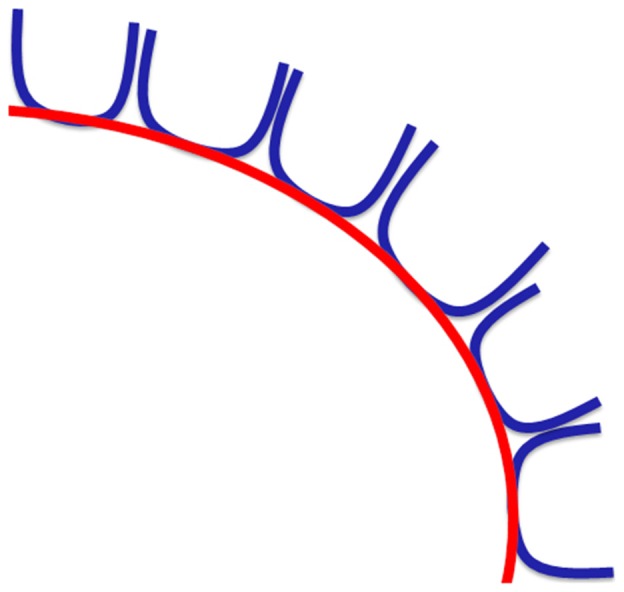
Hypothesis on how consecutive U-shaped fibers (in blue) can form a long pathway (in red).

FT shows that the SAF can be consistently reconstructed with FT. However, the presence of a long association fiber is not fully supported by dissection, which necessitates hypothesizing about why this pathway is still found with FT. An explanation could be that a series of consecutive U-shaped fibers that connect adjacent gyri emerge to form a long pathway (see Figures 8, 12). This possibility is supported by the work of Maldonado et al. (2012), where they propose that the dorsal component of SLF is primarily composed of U-shaped fibers. However, dissection also revealed a distinct WM structure with the same orientation as the proposed fiber bundle after the explicit removal of U-fibers. One may argue that the fibers are part of the dorsal component of the SLF system (i.e., SLF I), but considering the common anatomical definitions and the territories of termination of the SLF, it is unlikely the case. Figures 11C,D highlights the different and well-known components of the SLF. Important to note that this regions is challenging for FT and dissection as well due to the high number of crossings with the descending and ascending (i.e., vertical) pathways.

Independent investigations are still necessary to decide whether the suggested structure is indeed a new one or a component of an already defined tract. A possible description based on new nomenclature that it is part of the large dorsal system of horizontal fibers connecting frontal and parietal cortices as a branch of the mesial longitudinal system (MesLS; Mandonnet et al., 2018), which can be further divided into an inner branch, which is the cingulum *per se* and an outer branch, the SAF. Note, such an outer branch is close to what Wang et al. (2016) named as supracingulate or paracingulate pathways. Supplementary Figure S3 shows the population of the SAF map CSD based modeling and high b-value data along with the cingulum from the JHU-ICBM DTI based WM atlas (Mori et al., 2005; Wakana et al., 2007; Hua et al., 2008) demonstrating that the SAF is not part of the cingulum.

While the agreement between dissection and FT is imperfect, it is important to describe the structure and recognize its occurrence. First, as this is a consistent finding, other researchers will likely discover and investigate the presence of this FT structure. Second, investigating the structural properties of the sub-compartments can improve the understanding of the U-fibers and rule out false structures, which were accidentally reconstructed from U-fibers. Third, this adds to the discussion of the value of FT, thereby raising additional awareness of the pitfalls of FT (Jones and Cercignani, 2010; Parker et al., 2013; O’Donnell and Pasternak, 2015), and the concerns in the field of connectomics (Hagmann et al., 2010; Fornito et al., 2013; Zalesky et al., 2016).

In the following sections, we will discuss that our FT results are plausible from the point of dMRI-based tractography, place our results in the context of other FT and dissection studies, provide suggestions for future investigations, and stimulate the debate about the validity of dMRI-based FT.

### Diffusion MRI

Recent developments in acquisition and data processing boosted the reliability of dMRI and increased the inherently low accuracy of mapping WM pathways with FT. The investigations in solving crossing fibers were imperative, as the reported structure would not or hardly be detected since the main diffusion direction here is along the forceps (see Figure 2). However, one of the pitfalls of dMRI is that it is an indirect measure of the underlying WM pathways and reflects the net displacement of water molecules along all structures within a large voxel. Different structural architectures can lead to the same diffusion profile (Jbabdi and Johansen-Berg, 2011), making it hard to reconstruct unambiguously the intrinsic fiber arrangement.

#### Impact of Modeling

The choice of diffusion signal modeling was critical as the SAF cannot be revealed with DTI. The tract is formed mostly from locally non-dominant fiber populations. Therefore, it can be mapped only with techniques, which can resolve crossing fibers. The single location where the tract is locally dominant is the most anterior part with up-down orientation, as depicted in blue in Figures 1A–C, and is also the only location revealed by DTI based population level tract mapping shown in Supplementary Figure S1. Following this line of reasoning, yet a system of non-dominant, interwoven fiber bundles are to be found with the help of high data quality and advanced modeling.

Another example of mapping a challenging fiber population without any well-known underlying architecture is showcased in the MyConnectome project (Poldrack et al., 2015), featuring longitudinal, high quality MRI data of a single adult male. In a small region of the CC, an anterior–posterior oriented set of fibers are dominating locally, yet the left-right oriented CC fibers are still present and continuous. This rare finding is not expected in the CC, but is remarkably strong in that the front-back oriented diffusion was larger than the left-right orientated diffusion and, hence, changed the color in the DEC map from red to green. Similar to the SAF, it is difficult to investigate parts of pathways where the underlying FOD profiles exhibit non-dominant peaks tangential to the pathway of interest.

#### Tract Consistency

CSD tractography results obtained with low and high b-value data show a high similarity of 90% for the core of the SAF, which is outstanding given the fact that WM fiber bundles vary in their size and position, especially for this is a small and inferior bundle. Compared with other larger bundles, the similarity is in line with previously reported overlap values of >90% (cingulum), >90% (core CC), >75% (CC), and >75% (iFOF; Thiebaut de Schotten et al., 2011b).

The presence of the SAF across different data samples and acquisition settings is indicative that the results are not merely based on acquisition artifacts. As FODs are sharper, more and longer streamlines are found at higher b-values as this will aid the resolving power of the different fiber populations within a voxel. Although we can show the SAF in case of all the participants, finding the complete trajectory is not trivial. The SAF can be incomplete, for example, when the FOD only contains the main peak of the crossing fibers and does not contain the minor second peak. While we were able to determine the main part of the SAF configuration, the dorsal terminations were hard to obtain, which was also an issue during dissection. Future studies, especially at higher spatial and angular resolution, may alleviate this question.

### Validation

A conceptual limitation of FT (Jeurissen et al., 2017) is the inability to resolve the functionality and directionality of anatomical links, where the existence of a physical connection is necessary to complete the cortical functions. Such functional confirmation can arise from the use cortico-cortical (Matsumoto et al., 2004, 2006, 2012) or axono-cortical evoked potentials (Yamao et al., 2014; Mandonnet et al., 2016), generally obtained with invasive techniques involving implants or electrodes during surgery.

The PLI results show an overall pathway that can resemble the presented structure; however, it is not clearly one consistent pathway. A drawback of PLI, however, is that it cannot distinguish between differently oriented crossing fibers, making it hard to be unambiguous about the exact pathway.

On the one hand, the brain dissections in this work could not clearly verify the existence of the SAF. On the other hand, the dissection results could not rule out the existence of the SAF given the limitations of this approach, especially in regions where multiple fiber systems exhibit crossing configurations. The brain data used for the FT results in this work are obtained from healthy volunteers. Hence, *ex vivo* investigations (dissection and PLI) on the same brains were not feasible. As such, a direct comparison was not possible.

### Complexity of Fiber Bundles

Interaction between FT and brain dissection methods drives both the validation of tracts, but also the discovery of true fiber bundles, thereby increasing our understanding of the design of the brain’s architecture (Yeatman et al., 2014; Meola et al., 2015; De Benedictis et al., 2016; Wu et al., 2016b; Hau et al., 2017). Over the years, more and more (parts of) WM bundles were revealed using FT, which were previously undetectable with the formerly existing techniques (Jbabdi and Johansen-Berg, 2011) such as the lateral projections of the CC, IFOF or the Aslant fiber bundle (Thiebaut de Schotten et al., 2012). However, the validity of some of these tracts is still debated. Yeatman et al. (2014) used tractography to rediscover the vertical occipital fasciculus (VOF); a bundle that caused controversies among neuroanatomists in the 19th century. These tractography findings were further confirmed *via* dissection by Wu et al. (2016c). In this context, the identification of the SAF with FT raises similar concerns and fuels the ongoing debate about validation with other approaches.

Other researchers have also proposed new or redefined WM structures. Track density imaging (TDI) on 7T dMRI (Calamante et al., 2010, 2011, 2012) recently demonstrated finer details of thalamocortical connections (Choi et al., 2018) and revealed the fiber system of the septum pellucidum area (Cho et al., 2015). In both studies the super-resolution ability of the voxelwise fiber count was used to generate images with high anatomical contrast and therefore exposed the aforementioned bundles. However, due to the noise sensitivity of the technique, the validity of structures revealed by TDI remains an open question (Dhollander et al., 2012, 2014).

In addition to new bundles, several studies have shown subcompartments of known WM pathways, which are validated by histology. These multi-component bundles such as the uncinate fasciculus (Hau et al., 2017), iFOF (Sarubbo et al., 2013; Caverzasi et al., 2014; Hau et al., 2016; Wu et al., 2016a) and SLF (Makris et al., 2005; Kamali et al., 2014) consist of a complexity of pathways that together form a united bundle. De Benedictis et al. (2016) used a microdissection approach to reveal and validate the presence of both homotopic as well as heterotopic fibers in the anterior half of the CC.

The cingulum, which has a similar shape and is in close approximation of the SAF, is also a multi-component bundle. The cingulum bundle consists of many short fibers as well as longer fibers that together have many different connections (Bajada et al., 2017). The cingulum is usually depicted as a continuous structure using FT although thorough research is showing a division in at least three subparts, each with their own distinct diffusion metrics (Jones et al., 2013a; Wu et al., 2016b). Given the similarity with the cingulum and the knowledge that many bundles have complex and multicomponent fiber organization, it seems plausible that the SAF also has a multicomponent organization.

### Future Directions

This study describes a new pathway identified with dMRI based FT, called the SAF, which may have several implications in *in vivo* neuroimaging studies about structural brain connectivity. If the SAF is considered to be a genuine new brain pathway, further research is needed to better understand the origin and the function of this structure. Investigation of this area at higher spatial resolution could be one avenue as recent work has shown that this coincides with a better understanding of complex crossing fiber structures (Schilling et al., 2017). This approach would then ideally be combined with dissection. We can expect more, previously not shown and locally non-dominant structures to be discovered as Jeurissen et al. (2013) showed that most of the WM in the human brain contains multiple populations. Other imaging techniques, such as optical coherence tomography (Huang et al., 1991) or optogenetics (Deisseroth, 2010) may provide complementary information that could verify the existence of the SAF.

We observed a large variability in the cross-sectional area of the SAF and future analysis of the structure should also entail examining common properties related to microstructure, shape, demographics, and even changes in pathological conditions. Furthermore, it is well-known that the frontal lobe is generally a challenging region to investigate by most MRI methods, because of the susceptibility induced distortions, which are further emphasized by EPI sequences caused by the air-tissue interfaces of the sinuses.

In the past, we have seen examples in which part of anatomical knowledge is abandoned from the mainstream, e.g., in case of the VOF or the IFOF. Since the influential work of Andreas Vesalius (*De humani corporis fabrica*, 1543, Padua Italy) anatomists are drawing, dissecting and reconstructing WM pathways with increasing precision as technology advances. It would not be unexpected if the SAF could reemerge from textbooks of human brain anatomy in a similar way.

## Conclusion

We have proposed the existence of the SAF in the human brain, a new WM bundle identified with dMRI based FT. We showed that it is consistent within several cohorts, across different acquisition settings and processing strategies. However, there are still uncertainties about the true underlying anatomy of the structure as evidenced by our complementary PLI and brain dissection findings.

## Data Availability

All datasets generated for this study are included in the manuscript and the supplementary files.

## Ethics Statement

### Dissection (Paris)

The study was approved by the local ethics committee of Pôle Neurosciences of Lariboisière Hospital. This study complied with the ethical standards.

### Dissection (Trento)

Dissections were performed in the context of the Structural and Functional Connectivity Lab (SFC-Lab) Project, regularly approved by the Ethical Commitee of the APSS di Trento (authorization N° 1837 released on September 26th 2013 and renewed with amendment on September 7th 2018).

### PLI

The brains were taken from persons without history of neurologic or psychiatric disease, who donated their body for anatomical study. All brains were collected from the body donor program of the Institute of Anatomy at the Technical University Aachen (RWTH).

## Author Contributions

SD, AH, and AL: protocol design and data analysis. SD, AH, FCorr, MTS, SS, FCors, AB, LP, EM, and AL: data interpretation. FCorr, MTS, SS, FCors, AB, LP, and EM: neuroanatomy. HA: PLI. SD, AH, FCorr, MTS, SS, FCors, AB, LP, EM, HA, TP, and AL: manuscript preparation. AH, FCorr, MTS, SS, FCors, AB, LP, MV, DJ, EM, HA, JE, TP, and AL: manuscript revision. AL: principal investigator.

## Conflict of Interest Statement

The authors declare that the research was conducted in the absence of any commercial or financial relationships that could be construed as a potential conflict of interest.
