# Supplementary material for: The Superoanterior Fasciculus (SAF): A Novel White Matter Pathway in the Human Brain?
Source: Front Neuroanat. 2019 Mar 5;13:24. doi: 10.3389/fnana.2019.00024 (PMC6412356; doi:10.3389/fnana.2019.00024)
Supplement: Supplementary file 1 [file Data_Sheet_1.docx]

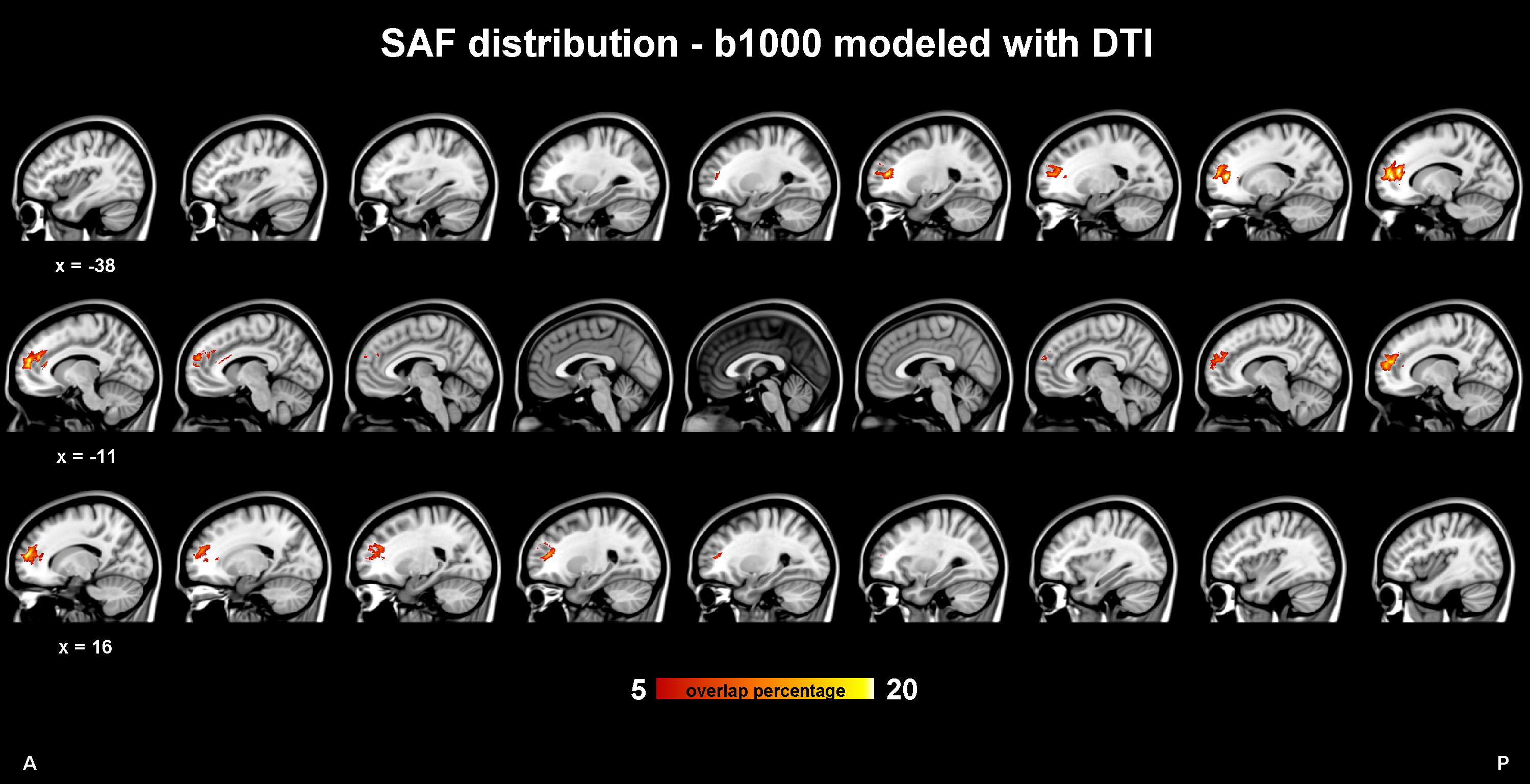


Supplementary Figure 1

Tract probability map in 1mm MNI stereotaxic space from 409 subjects of the HCP cohort in sagittal view. DTI estimation with REKINDLE was used on the low b-value data. Note the narrower range of the tract mask overlap for better contrast. The % indicates the fraction of subjects for which the SAF is present in a given voxel.


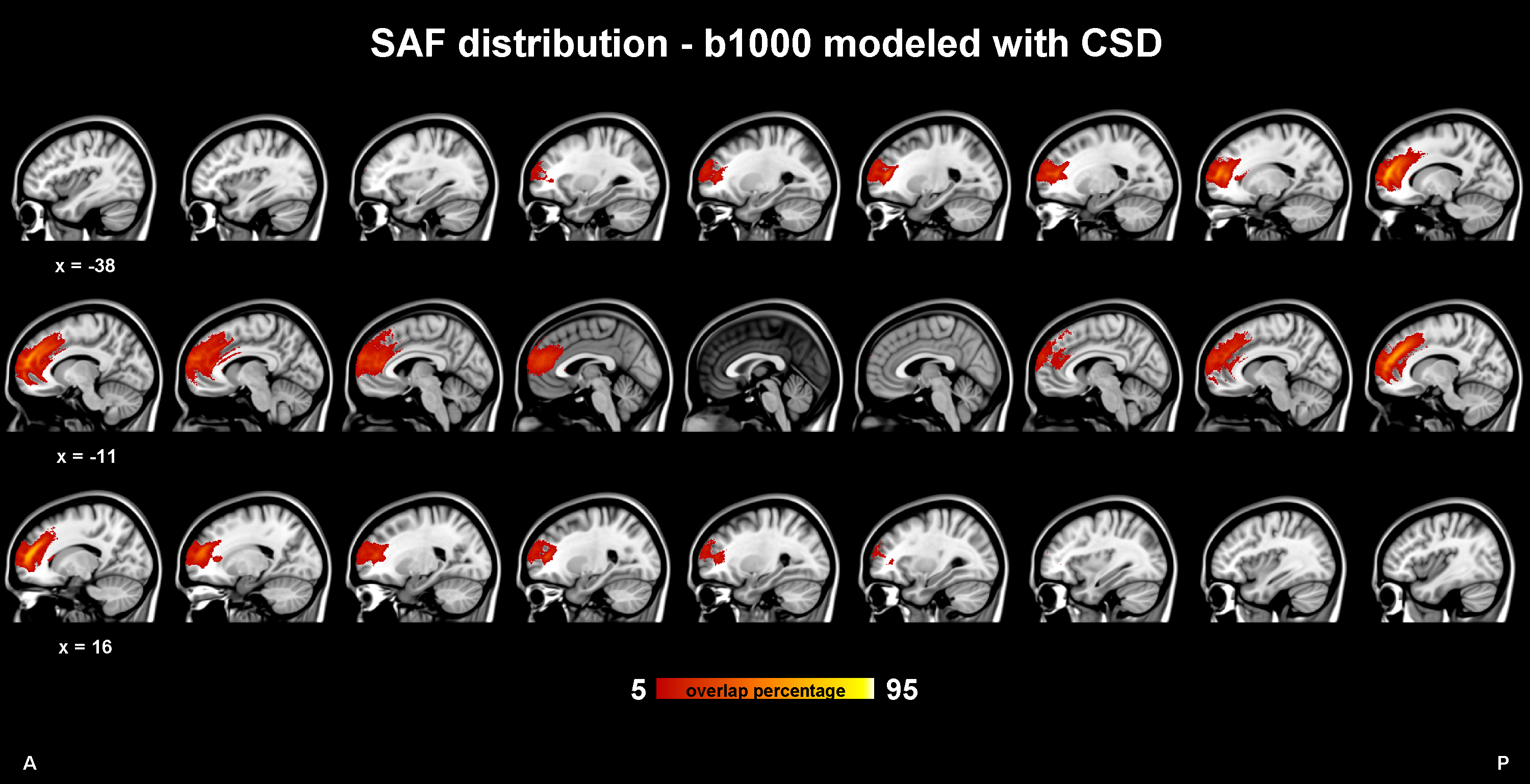


Supplementary Figure 2

Tract probability map in 1mm MNI stereotaxic space from 409 subjects of the HCP cohort in sagittal view. CSD modeling with recursive calibration was used on the low b-value data. The % indicates the fraction of subjects for which the SAF is present in a given voxel.


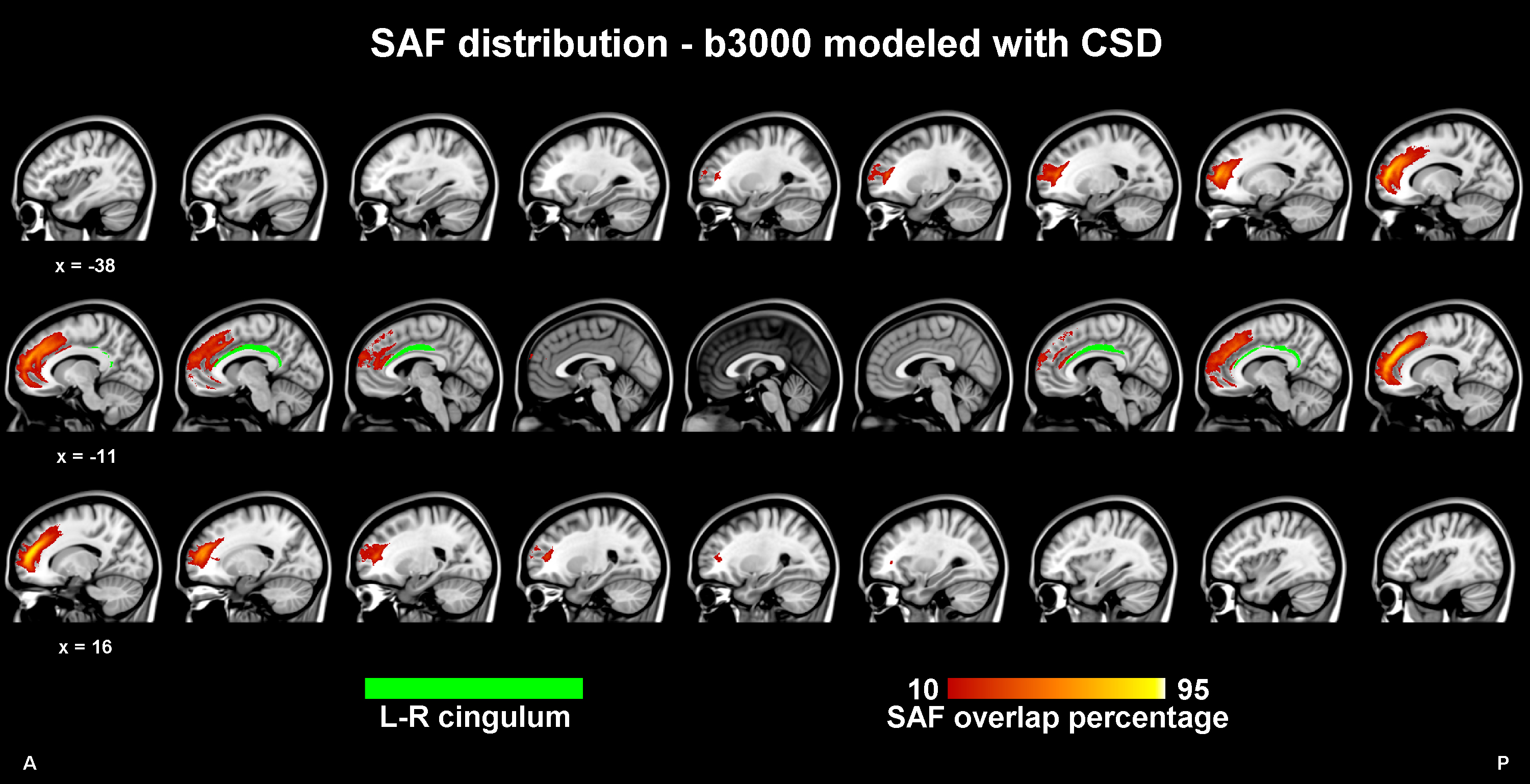


Supplementary Figure 3

Tract probability map in 1mm Montreal Neurological Institute (MNI) stereotaxic space from 409 subjects of the HCP cohort in sagittal view. CSD modeling with recursive calibration was used on the high b-value data. The % indicates the fraction of subjects for which the SAF is present in a given voxel. Green shows the left and right cingulum labels from the JHU DTI-based white-matter atlas for anatomical reference.
